# Opportunities to address lung cancer disparities among African Americans

**DOI:** 10.1002/cam4.348

**Published:** 2014-09-14

**Authors:** Steven S Coughlin, Patricia Matthews-Juarez, Paul D Juarez, Courtnee E Melton, Mario King

**Affiliations:** 1 Research Center on Health Disparities, Equity, and the Exposome, University of Tennessee College of MedicineMemphis, Tennessee; 2 Department of Preventive Medicine, University of Tennessee Health Science CenterMemphis, Tennessee

**Keywords:** African Americans, cancer survivorship, cigarette smoking, health status disparities, lung cancer, prevention

## Abstract

Race and socioeconomic status are well known to influence lung cancer incidence and mortality patterns in the U.S. Lung cancer incidence and mortality rates are higher among blacks than whites. In this article we review opportunities to address disparities in lung cancer incidence, mortality, and survivorship among African Americans. First, we summarize recent advances in the early detection and treatment of lung cancer. Then we consider black-white disparities in lung cancer treatment including factors that may contribute to such disparities; the literature on smoking cessation interventions for patients with or without a lung cancer diagnosis; and the important roles played by cultural competency, patient trust in their physician, and health literacy in addressing lung cancer disparities, including the need for culturally competent lung cancer patient navigators. Intervention efforts should focus on providing appropriate quality treatment for lung cancer and educating African Americans about the value of having these treatments in order to reduce these disparities. Culturally competent, patient navigation programs are needed that support lung cancer patients, especially socioeconomically disadvantaged patients, from the point of diagnosis to the initiation and completion of treatment, including cancer staging.

## Introduction

Lung cancer is the leading cause of cancer-related death among both men and women in the U.S. and many other countries. In 2013, there were an estimated 228,190 cases of lung cancer in the U.S. and 159,480 deaths from the disease [1]. Non-small cell lung cancer (NSCLC) accounts for about 85% of lung cancer in the U.S. Race and socioeconomic status are well known to influence lung cancer incidence and mortality patterns in the U.S [2–4]. During 2007–2011, lung cancer incidence rates in the U.S. were higher among blacks than whites [3].

Challenges experienced by economically disadvantaged African Americans in receiving lung cancer services include: locations of diagnostic and treatment facilities outside of the patient's neighborhood, transportation, lack of understanding of lung cancer, unavailability of support resources for them to access lung cancer treatment services, lack of access to primary care, and lack of familiarity with resources available from cancer support organizations. Factors that likely contribute to the relatively high lung cancer death rates for African Americans include the roles played by low health literacy, lack of knowledge about lung cancer, attitudes, beliefs, cultural factors, and lack of access to services. Other barriers to receiving lung cancer services include fear and mistrust, uncertainty, lack of information, lack of a primary care provider, and unfamiliarity with providers. These challenges are worsened by the disjointed, categorical nature of service provision and the lack of a coordinated effort to address lung cancer treatment challenges in many communities. Although genetic and epi-genetic factors may also have a role in black-white disparities in lung cancer, studies of racial differences in the frequency of epidermal growth factor receptor mutations in African American and Caucasian patients with NSCLC have had inconsistent results [5, 6].

In this article, we review opportunities to address disparities in lung cancer incidence, mortality, and survivorship among African Americans. First, we summarize recent advances in the early detection and treatment of lung cancer. Then we consider black-white disparities in lung cancer treatment including factors that may contribute to such disparities; the literature on smoking cessation interventions for patients with or without a lung cancer diagnosis; and the important roles played by cultural competency, patient trust in their physician, and health literacy in addressing lung cancer disparities, including the need for culturally competent lung cancer patient navigators. Finally, we discuss remaining challenges in this area and summarize findings from this review.

## Recent Advances in the Early Detection and Treatment of Lung Cancer

The prognosis for NSCLC is poor with 5-year survival rates ranging from 49% for patients with stage 1A disease to about 1% for those with stage IV [7]. Receipt of timely, stage-appropriate care for patients with NSCLC can increase the length of survival [7, 8]. Once a patient has been staged, timely receipt of surgical resection has an important impact on survival outcomes among those with early-stage NSCLC. Surgical resection remains the primary and preferred approach to the treatment of stage I and II NSCLC [9, 10]. The use of adjuvant chemotherapy for stage II NSCLC is recommended and has shown benefit. Every patient should have systematic mediastinal lymph node sampling at the time of curative intent surgical resection. Perioperative morbidity and mortality are reduced and long-term survival is improved when surgical resection is performed by a board certified thoracic surgeon [9]. In the U.S., about 30% of pulmonary resections are performed by general surgeons. Recommended treatment for patients with late-stage disease ranges from chemotherapy and radiation with or without surgery (stage IIIA), to chemotherapy and radiation without surgery or chemotherapy alone (stage IIIB), to chemotherapy alone for patients with metastatic disease (stage IV).

Epidermal growth factor receptor mutation (EGFR) testing is increasingly performed using biopsy material from lung cancer patients, as targeted tyrosine kinase inhibitor drugs have been introduced for the treatment of patients with advanced NSCLC. These targeted therapies have been shown to improve progression-free survival and quality of life in patients with high EGFR expression in their tumors, as compared with platinum-based chemotherapy [11].

In the National Lung Screening Trial (NLST), 53,454 older current or former heavy smokers were randomized to receive low-dose computed tomography (LDCT) or chest radiography for three annual screens [12]. LDCT detected more than twice the number of early-stage lung cancers and resulted in a stage shift from advanced to early-stage disease. Persons screened with LDCT had a 20% relative reduction in lung cancer mortality as compared with those screened with annual chest X-rays. The trial results have not been reported by race. Most of the individuals randomized to the trial (48,549, or 91%) were white and only 2378 (4.4%) were black.

The probability of cancer in screen-detected nodules depends on their size and whether the nodules are detected on prevalence or incidence screens [13]. Management strategies for screen-detected nodules include serial CT imaging and CT-guided biopsy for larger nodules and those that demonstrate growth on follow-up.

Although the NLST has increased interest in LDCT screening for lung cancer among higher risk persons, additional work remains to be done. For example, there is a need for additional studies of patient acceptance of lung cancer screening [14]. In addition, some primary care providers may not be convinced of the efficacy of lung cancer screening or that the benefits outweigh the risks. Guidelines have been proposed to help identify an appropriate screening population and to develop standards for radiological testing. Challenges with routine lung cancer screening include the potential for overdiagnosis and the large number of false-positive results. In the NLST, 24.2% of the screens were positive, and 96.4% of these proved to be false-positive results [15]. Although many questions remain about LDCT screening, a comprehensive lung cancer screening program aimed at higher risk persons can increase detection of potentially curable disease and introduce a new model of lung cancer surveillance and management [12, 16]. Some oncology and cancer advocacy organizations, including the National Comprehensive Cancer Network and the American Cancer Society, have published guidelines for LDCT screening and a few of the major insurance providers now pay for LDCT screening in higher risk persons [12, 17].

## Black-White Disparities in Lung Cancer Treatment

The survival of patients with stage I, II lung cancer differs significantly based upon race/ethnicity and socioeconomic status. Racial differences in cancer outcomes may be due to several factors including decreased access to quality care, differences in tumor biology resulting in increased aggressiveness or resistance to treatment, socioeconomic factors influencing treatment options, increased comorbid conditions among African Americans, and suboptimal patient-physician interactions [18, 19]. Several studies have identified racial disparities in the quality of care among NSCLC patients that may contribute to racial differences in outcomes [7]. For example, racial disparities in lung cancer staging with positron emission tomography have been observed, even though proper staging is crucial for effective treatment planning [20]. Racial disparities in the timeliness of care in lung cancer patients have also been observed [21]. Numerous studies have shown that African American patients are less likely to receive surgical resection than whites [8, 22–25]. African Americans appear to have the lowest survival for early-stage NSCLC [7]. Native American Indians and Native Alaskans have worse survival than non-Hispanic whites as well [9].

Shugarman et al. [24]. evaluated the relationship between race and sex with timely and appropriate treatment for NSCLC treatment using surveillance epidemiology and end results (SEER) data linked to Medicare claims data over the period of 1995 to 1999 (*n* = 22,145). Blacks were 66% less likely to receive timely and appropriate treatment than whites. Black men were least likely to receive resection (22% compared with 43.7% for white men). Blacks were 34% less likely to receive timely surgery, chemotherapy, or radiation for stage III disease and were 51% less likely to receive chemotherapy in a timely fashion for stage IV disease relative to whites [24]. Similar findings were reported by Hardy et al. [8]. who examined racial disparities in treatment for NSCLC using SEER data linked to Medicare claims data for the period 1991 to 2002 (*n* = 83,101 patients aged ≥ 65 years). Steele et al. [25]. linked data from the Alabama State Cancer Registry with state Medicare data to examine urban/rural patterns in receipt of treatment for NSCLC among black and white Medicare beneficiaries in Alabama. They identified 3481 cases of stages I-IV and unknown stage NSCLC diagnosed from 2000–2002. Among those with resectable NSCLC (stages I–IIIA), urban whites were more likely to undergo surgical resection than urban blacks (49.3% vs. 33.0%, respectively), and more rural whites than rural blacks (49.8% vs. 23.9%, respectively) underwent surgery. There was less variation by race and urban/rural residence in the receipt of chemotherapy and radiation therapy. The authors noted that future studies should explore access to care and patient perceptions about treatment [25].

The causes of these racial differences are complex and include patient, environmental, and health system factors [7]. When patients receive appropriate care at the right time, few racial differences in NSCLC survival rates occur. Recent studies of NSCLC patients who received treatment at Veterans Affairs facilities, a single-payer, accessible health care system, have demonstrated few black-white disparities in lung cancer outcomes [7, 26]. Nevertheless, there is ample evidence that pronounced lung cancer disparities persist in the U.S. Black-white differences in patient refusal rates, as well as patient attitudes, beliefs, and knowledge about lung cancer, may contribute to racial differences in receipt of appropriate treatment for NSCLC [22, 27]. Patient attitudes such as fatalism and denial can lead to delays in presenting for medical evaluation when symptoms occur [12]. In addition, important differences have been identified in the understanding of smoking-related risks and lung cancer among different racial/ethnic and socioeconomic groups. Individuals from disadvantaged backgrounds are more likely to have misperceptions about their risk of lung cancer, the benefits of surgical resection, and lung cancer mortality [12, 28–30]. Higher educational attainment and higher economic status are associated with greater understanding of the state of the science on smoking and lung cancer [31–33].

## Cigarette Smoking and Other Risk Factors for Lung Cancer

Cigarette smoking is the most important preventable cause of lung cancer, although second hand smoke, occupational exposures (e.g., asbestos, chromium, diesel exhaust, some forms of silica), ionizing radiation, and genetic factors also contribute to lung cancer morbidity and mortality [34]. Exposure to radon gas is a leading cause of lung cancer in nonsmokers.

In the U.S., cigarette smoking causes about 90% of lung cancers. According to the CDC, 19.7% of American adults who are white were current smokers in 2012 as compared with 18.1% of American adults who are black. African Americans are more likely to smoke mentholated cigarettes. Using other tobacco products such as cigars or pipes also increases risk of lung cancer. According to the CDC, tobacco smoke is a toxic mix of more than 7000 chemicals. At least 70 are known to cause cancer. Individuals who smoke are 15–30 times more likely to get lung cancer or die from lung cancer than people who do not smoke. The risk of lung cancer increases with a greater number of years a person smokes and the number of cigarettes smoked each day.

The USPHS Clinical Practice Guideline for the Treatment of Tobacco Dependence provides best practice standards for treating tobacco dependence [35]. Techniques stemming from behaviorally based counseling models, including motivational enhancement and skills training, have been found to be effective for smoking cessation [36]. The provision of social support is also helpful. Pharmacotherapies for smoking cessation include nicotine replacement therapy (nicotine patch, gum, inhaler, spray, and lozenge) and the antidepressant bupropion. A wide variety of evidence-based public health and clinical interventions are available to help people quit smoking, as systematically reviewed by the Guide to Community Preventive Services and the U.S. Preventive Services Taskforce (http://www.thecommunityguide.org/index.html; http://www.uspreventiveservicestaskforce.org/recommendations.htm). Culturally appropriate interventions have been developed to help African Americans stop smoking [37–42]. Other interventions are especially well-suited for patients who have been diagnosed with lung cancer or other tobacco-related illness [36, 43, 44]. However, there is currently a paucity of evidence-based, culturally appropriate interventions designed to assist African American lung cancer patients quit smoking.

Approaches for treating cancer patients of all races who smoke include countering fatalistic attitudes by informing patients of the short term benefits of quitting smoking related to their cancer treatment and about the long term benefits related to increased survival [45, 46]. As patients in general are often particularly sensitive to any perceived blame for a smoking-related illness, providers should explain the strong role of nicotine addiction and facilitate motivation for behavioral change using the social support of family, friends, and healthcare professionals [36]. Education about the health risks of continuing to smoke after a cancer diagnosis and the health benefits of smoking cessation can be useful in increasing patient motivation and interest in quitting. Providers can assess a smoker's confidence (self-efficacy) in quitting and then tailor the intervention accordingly. Cancer patients who smoke can be referred to the 1-800-QUIT-NOW national telephone quit line offered by each state. The smoker is assigned a personal coach and can have several individualized counseling sessions.

Following a cancer diagnosis, continued tobacco use increases cancer treatment toxicity, recurrence, second primary tumors, and mortality, and impairs quality of life [36]. In a study of over 20,000 pulmonary, gastrointestinal, and urologic patients with cancer, current smoking increased the risk of pulmonary complications, surgical site infection, and 30-day mortality after surgery [47]. For the major cancer treatment modalities (surgery, chemotherapy, and radiation therapy), smoking has been found to diminish treatment effectiveness, increase side effects, and interfere with wound healing [36]. Cigarette smoking is an established risk factor for a variety of pulmonary, cardiovascular, and infectious complications [48]. Nevertheless, studies have shown that 50–83% of cancer patients continue to smoke after a diagnosis [46]. Some cancer patients may accept the negative consequences of smoking or not see the point in quitting smoking. Depression is common among cancer patients and people who are depressed are more likely to use tobacco and they often have a harder time quitting [49, 50].

There is a need for increased access to tobacco cessation support for cancer patients and to study ways to increase the efficacy of tobacco cessation after a cancer diagnosis [51]. There is a paucity of studies that have combined provider training on tobacco cessation with training on cultural competency and patient-provider communication. Improving access to tobacco cessation support has been sparsely studied in the oncology setting compared with the general population. However, studies completed so far indicate that cancer patients are receptive to evidence-based tobacco cessation guidelines [51].

Unfortunately, studies suggest that many oncology providers do not provide regular assistance to cancer patients to stop smoking [52, 53]. A randomized trial of usual care versus physician led cessation for cancer patients found that 56% of the physicians recommend quitting to their patients, but only 35% discuss health benefits of quitting, 5% help to set a quit date, 17% provide materials to help quit, and 19% provide a nicotine prescription to quit [54]. A survey of nearly 1200 members of the American Society for Clinical Oncology found that about 90% of oncologists believe tobacco use affects cancer outcome and that cessation support should be provided to cancer patients, but only 40% provide assistance to help patients quit smoking [52]. Even though many health care providers feel that more should be done to assist patients to stop smoking, they may lack confidence or training in how to provide smoking cessation services. Physicians who care for lung cancer patients may be pessimistic about their ability to help patients stop using tobacco or have concerns about patient resistance to treatment [52]. Training health professionals about smoking cessation has been shown to increase delivery of these services and to increase quit rates [55]. Resources that are available to train providers include pocket guides, online material, and the 5 A's system of counseling patients to stop using tobacco (Ask, Advise, Assess, Assist, and Arrange) [35]. The latter strategies include: (1) identifying and documenting tobacco use for every patient at every visit, (2) strongly urging every tobacco user to quit, determining the willingness of the tobacco user to make a quit attempt, using counseling and pharmacotherapy to aid patients in quitting, and scheduling follow-up contact [35, 36]. Potential barriers to proper provider education include overloaded curricula, low priority of tobacco control content, and negative attitudes toward tobacco control [46]. In addition, cancer patients who are uninsured may not be able to afford cessation medications, although most insurers now cover tobacco cessation treatments [46]. As a result of these issues, many cancer patients are not receiving appropriate tobacco cessation support and the diagnosis of cancer is underused as a teachable moment for smoking cessation [36, 51].

## Cultural Competency, Patient Trust in their Physician, and Health Literacy

Patient's trust in their physician is essential for desirable treatment outcomes such as satisfaction and adherence [56]. This is especially true in oncology due to the life threatening nature of cancer. Studies have shown that patient trust is enhanced by the physician's technical competence, honesty, and patient-centered behavior. A trusting relationship between a patient and their physician can result in improved communication and medical decision-making, decrease patient fear, and improve treatment adherence [56]. Perceived quality of lung cancer communication has been associated with receiving potentially curative surgery for early-stage disease (Dalton et al. [57].). In a study of decision making in early-stage NSCLC patients seen at 5 academic and community medical centers (*n* = 386), Dalton et al. [57]. found that income and trust score were significantly associated with an overall communication scale. Gordon et al. [58]. examined black-white differences in patient trust associated with physician-patient communication about lung cancer treatment. Data were obtained from 103 patients seen at thoracic surgery or oncology clinics in a large Veterans Affairs hospital in the southern U.S. for initial treatment recommendation for suspicious pulmonary nodules or lung cancer. Black patients had lower post-visit trust in their physician than white patients (*P* = 0.02). Compared with white patients, black patients judged the physicians' communication as less informative, less supportive, and less partnering. The authors noted that their findings raise concern that black patients may have lower trust in their physician in part because of poorer physician-patient communication [58, 59]. Improving patient communication with their provider is essential for ensuring that patients receive optimal care for NSCLC [57]. Studies have shown that effective patient-physician communication is related to improved adherence to medical regimens, better decision making, and increased satisfaction with the patient-physician relationship [60, 61]. Cultural competency skills can assist patient-provider communication.

Cultural competency influences how health messages are transmitted and perceived, how illness is defined, how symptoms are described, when and where care is obtained, and how treatment options are considered. Cultural competency, which can be taught as part of medical education and continuing professional education programs, includes the acquisition and integration of knowledge, with awareness, attitude, and skills about culture and cultural differences that enables health care professionals to provide optimal care to patients from different racial, ethnic, socioeconomic, and cultural backgrounds. Matthews-Juarez and Weinberg [62] define culture as the integration of patterns of human behavior that includes language, thoughts, communications, behaviors, customs, mores, beliefs, values, and institutions of different racial/ethnic or social groups.

Patient health literacy is also important. Low health literacy has been associated with decreased use of preventive services such as smoking cessation programs, increased risk of having a chronic disease such as cancer, increased use of emergency services, poorer treatment adherence, and poorer health outcomes [63]. The Institute of Medicine defines health literacy as “the degree to which individuals have the capacity to obtain, process, and understand basic health information and services needed to make appropriate health decisions [64].” Low health literacy is associated with decreased likelihood of seeking cancer information from a health care professional, increased sense of fatalism about cancer, decreased participation in cancer control programs, and later stage at diagnosis [65]. Health literacy also influences patient-provider communication. Individuals with low health literacy are more passive when interacting with providers, less likely to engage in shared decision making, and are less likely to ask questions [66]. The ability to effectively communicate with providers is particularly important due to the complexity of cancer care. Furthermore, cancer prevention and control messages are often written at too high a reading level for individuals with marginal literacy skills, and health professionals often overestimate the health literacy skills of patients, possess inadequate awareness about health literacy issues, and do not routinely use recommended communication strategies [67, 68]. Although studies have suggested that African Americans in the United States have lower health literacy than their Caucasian counterparts, racial differences in health literacy may be due to uncontrolled confounding by other factors such as age and education.

Differences in the quantity and quality of health care provided to African American men and women are critical to understanding cancer disparities. This includes the nature and quality of cultural competency interventions. A number of different constructs have been utilized as potential targets of cultural competency interventions designed to increase access to care and decrease health disparities in racial/ethnic or disadvantaged groups [69, 70]. These interventions should include methods for defining and operationalizing social constructs (culture, family, and community) as well as measuring behavior-related constructs associated with culture including psychosocial factors such as fear, isolation, fatalism, trust, and respect [71].

## Lung Cancer Patient Navigators

Patients who are newly diagnosed with lung cancer are not only coping with the emotional trauma of a cancer diagnosis, they are also expected to digest complicated and often threatening information about their illness and treatment procedures [61]. Many cancer patients, especially those who are socioeconomically disadvantaged, leave their health care visit confused about their diagnosis, prognosis, options for treatment, and next steps [72]. The challenges of successfully navigating the health care system can conceivably be overcome with the assistance of patient navigators (e.g., oncology nurses) who are trained to help patients through the processes of care. The linkage of patient navigators with patients diagnosed with lung cancer beginning at the point of diagnosis, could help ensure treatment completion and improve survival rates. Potential benefits include a reduction in fear of cancer, increased trust in oncologists and other health care providers, improved patient satisfaction, increased knowledge of lung cancer treatment options, and better adherence and completion of treatment regimens. Studies involving women with breast cancer, colorectal cancer patients, and men and women with lung cancer, have demonstrated that cancer patients who have patient navigators that provide services that are culturally appropriate, confidential, respectful, and compassionate, can experience better outcomes. There have been few studies of patient navigators for lung cancer patients and there is a paucity of nurse patient navigator interventions that are culturally appropriate for low income African American lung cancer patients.

Patient navigation includes support and guidance offered to vulnerable persons with a lung cancer diagnosis, with the goal of facilitating receipt and completion of timely and appropriate staging and treatment and maximizing quality of life [73]. Patient navigators can assist minority patients and other patients who are unfamiliar with the cancer center structure. The patient navigator model is based on the care management or case management model, which has 4 components: (1) case identification, a systematic approach to the identification of individuals newly diagnosed with lung cancer who are in need of follow-up care; (2) identifying individual and institutional barriers to receiving care; (3) developing an individualized plan to address the barriers that are identified; and (4) tracing each case with a systematic method of follow-up, through the completion of treatment. Patient navigators can provide emotional and support services to patients and their families and assist them with the coordination of care among health care and other community service care providers.

In order to help address disparities in lung cancer, navigators should not only be knowledgeable about cancer diagnosis and treatment, but also understand barriers to care, communication skills, cultural competency, and local networks of resources to support patients. Nurse patient navigators can explain why follow-up tests (biopsy or CT) are needed and coordinate scheduling; be available to speak with the patient and his or her family about a cancer diagnosis, provide lung cancer education, including general information about the nature of various treatment options, and assist with appointment scheduling once the treatment process has been decided upon by the patient and his or her physician.

## Discussion

Disparities in lung cancer among African Americans should be understood within the context of disparities among other groups identified by race, ethnicity, culture, and place of residence. It has long been recognized that pronounced health disparities exist across population groups defined by race/ethnicity, socioeconomic factors, urban versus rural residence, and other factors. In addition to lung cancer, African Americans and some other racial/ethnic minority populations are 1.5–2.0 times more likely than whites to have most of the major chronic diseases. Chronic diseases are more common among socioeconomically disadvantaged persons. Although decades-long efforts have been made to study the determinants of health disparities and to identify effective ways to address these inequities, health disparities and inequalities have been remarkably persistent. Although the root causes for health disparities are complex, well-developed, evidence-based approaches exist to address them and new research is likely to identify even more solutions. Improving the cultural competence of health care providers and the health care system are examples of existing evidence-based approaches for reducing health disparities.

Moving a patient through a lung cancer care/cancer continuum is a complex and individualized process. Several studies have shown that after a lung cancer diagnosis, many men and women, particularly African Americans, do not receive treatment consistent with clinical guidelines for lung cancer staging and treatment. People who are least likely to navigate the health care system for lung cancer often have a low level of education, less health literacy, belong to a racial/ethnic group, are uninsured/underinsured, poor, and live in a medically underserved community. Cancer care is often fragmented, inadequately coordinated, and not always organized around the needs of the patient. Culturally competent, patient navigation programs are needed that support lung cancer patients, especially socioeconomically disadvantaged patients, from the point of diagnosis to the initiation and completion of treatment, including cancer staging.

Health care providers have an important role to play in both the primary prevention of lung cancer and other smoking-related diseases, and in the tertiary prevention of lung cancer recurrence and other tobacco related malignancies among lung cancer patients. Such efforts can help to address disparities in lung cancer among African Americans. Resources are available such as the USPHS Clinical Practice Guideline for the Treatment of Tobacco Dependence [35] and the National Cancer Institute's How to Help Your Patients Stop Smoking. Tobacco cessation techniques stem from behaviorally based counseling models such as motivational enhancement and skills training [36]. Cultural competency and patient-provider communication training are likely to be key for providers to help African American patients stop smoking. Providers should inform their patients about resources such as the 1-800-QUIT-NOW national telephone quit line. First line pharmacotherapies for smoking cessation include nicotine (patch, gum, inhaler, spray, and lozenge) and bupropion.

As lung cancer and other diseases become more preventable due to advances in medical knowledge, individuals with greater access to resources tend to benefit more, which can lead to the worsening of health disparities [4]. Public health interventions are therefore needed to facilitate a more equitable distribution of medical advances and improved uptake and utilization of lung cancer treatment among lower socioeconomic groups such as economically disadvantaged African Americans and other at-risk populations.
